# Ultrasound guided needle biopsy of axilla to evaluate nodal metastasis after preoperative systemic therapy in cohort of 106 breast cancers enriched with *BRCA1/2* pathogenic variant carriers

**DOI:** 10.1186/s13053-021-00187-w

**Published:** 2021-07-07

**Authors:** Baiba Līcīte, Arvīds Irmejs, Jeļena Maksimenko, Pēteris Loža, Genādijs Trofimovičs, Edvīns Miklaševičs, Jurijs Nazarovs, Māra Romanovska, Justīne Deičmane, Reinis Irmejs, Gunta Purkalne, Jānis Gardovskis

**Affiliations:** 1 Department of Surgery, Riga Stradiņš University, Pilsoņu iela 13, LV-1002 Riga, Latvia; 2 Department of Surgery, (Affiliated Partner of the European Reference Network on Genetic Tumour Risk Syndromes (ERN GENTURIS)), Pauls Stradiņš Clinical University Hospital, Pilsoņu iela 13, LV-1002 Riga, Latvia; 3 Institute of Oncology, Riga Stradiņš University, Pilsoņu iela 13, LV-1002 Riga, Latvia; 4 Department of Pathology, Pauls Stradiņš Clinical University Hospital, Pilsoņu iela 13, LV-1002 Riga, Latvia; 5 Department of Radiology, Pauls Stradiņš Clinical University Hospital, Pilsoņu iela 13, LV-1002 Riga, Latvia; 6 St John’s College, University of Cambridge, Cambridge, England; 7 Department of Oncology, Pauls Stradiņš Clinical University Hospital, Pilsoņu iela 13, LV-1002 Riga, Latvia

**Keywords:** Breast cancer neoadjuvant axilla cytology *BRCA1/2*

## Abstract

**Background:**

Aim of the study is to evaluate the role of ultrasound guided fine needle aspiration cytology (FNAC) in the restaging of node positive breast cancer after preoperative systemic therapy (PST).

**Methods:**

From January 2016 – October 2020 106 node positive stage IIA-IIIC breast cancer cases undergoing PST were included in the study. 18 (17 %) were carriers of pathogenic variant in *BRCA1/2*. After PST restaging of axilla was performed with ultrasound and FNAC of the marked and/or the most suspicious axillary node. In 72/106 cases axilla conserving surgery and in 34/106 cases axillary lymph node dissection (ALND) was performed.

**Results:**

False Positive Rate (FPR) of FNAC after PST in whole cohort and *BRCA1/2* positive subgroup is 8 and 0 % and False Negative Rate (FNR) – 43 and 18 % respectively. Overall Sensitivity − 55 %, specificity- 93 %, accuracy 70 %.

**Conclusion:**

FNAC after PST has low FPR and is useful to predict residual axillary disease and to streamline surgical decision making regarding ALND both in *BRCA1/2* positive and negative subgroups. FNR is high in overall cohort and FNAC alone are not able to predict ypCR and omission of further axillary surgery. However, FNAC performance in *BRCA1/2* positive subgroup is more promising and further research with larger number of cases is necessary to confirm the results.

## Background

According to recent studies pathological complete response (pCR) after neoadjuvant chemotherapy (NAC) in node positive breast cancer is observed in up to 40–75 % of cases [1–4]. *BRCA1* positive breast cancer subgroup also has high rate of pCR – up to 61 % [5]. It means that preoperative systemic therapy has completely eliminated all regional cancer involvement and total removal of all axillary lymph nodes is not justified. However, until recently axillary lymph node dissection (ALND) was performed in all node positive cases irrespective of response to PST. According to latest NCCN guidelines for breast cancer, conservation of axilla should be considered, if nodes are clinically negative after PST, but optimal axillary management after PST is still not known [6, 7]. Several preoperative and intraoperative axillary reevaluation approaches have been studied recently including physical examination, imaging (e.g. ultrasound, PET-CT) and biopsy techniques [8, 9]. Imaging studies of PET/CT is controversial for axillary staging after PST [10]. Combined clipped and sentinel node biopsy approach has very low false negative rate 1,4 %, but disadvantage is necessity of intraoperative frozen section and/or potential for repeated axillary surgery in cN2 and cN3 cases [9].

FNAC or core needle biopsy has been widely used for initial evaluation of axillary nodal status in case of breast cancer, but to the best of our knowledge there are only two small series (including our earlier one), which are reporting on axillary lymph node FNAC accuracy after PST [11, 12]. Aim of the study is to further evaluate the role of FNAC in the restaging of node positive breast cancer after PST, including subgroup of *BRCA1/2* positive cases.

## Materials and methods

Prospective cohort study was carried out at the state tertiary healthcare institution. This study was approved by the Pauls Stradiņš Clinical Univerity hospital Development Society Clinical Research Ethics Committee (310816-12 L) and Riga Stradiņš University Research Ethics Committe (72/29.10.2015.) All participants provided written informed consent.

From January 2016 – October 2020 106 FNAC confirmed node positive stage IIA-IIIC breast cancer cases undergoing PST were recruited to the study, including 18 (17 %) carriers of pathogenic variant in *BRCA1* (2- 300T > G, 5- 4154delA, 7-5382insC, 2- c.5117G > A, 1- del exon20) and *BRCA2* (1- 9097delA). All patients were female with median age of 51 years (range 25–75 years).

18/106 (17 %) of cases belonged to HER2 positive subtype, 24/106 (23 %) were triple negative (TN) and 64/106 (60 %) Luminal. 17/106 (16 %) cases were Luminal HER2 positive. Cases are considered as TN if HER2 is negative and estrogen/progesteron is < 10 %. 100/106 (94 %) had ductal, 5/106 (5 %) cases - lobular and 1/106 (1 %) combined pathology.

According to study protocol both before and after PST fine needle aspiration (FNA) was performed if at least one lymphnode on ultrasound had cortex > 3mm or absence of fatty hilum was present irrespective of lymphnode size. In case of several lymphnodes with the suspicious features, one with the most prominent changes and/or the most accessible from technical perspective was chosen.

FNA procedure was performed following the technique described by Dusenbery, 1997. Once the needle was in lymphnode, suction was applied to the syringe and the needle tip was moved back and forth within the node. On average 10 to 20 excursions with the needle were performed before obtaining material in the hub of the needle.

In case of malignant finding in cytology, marker was introduced in affected lymphnode to facilitate restaging and targeted surgery of axilla. Initially V-mark™ Breast Biopsy Site Marker with Titanium Anchor (Argon Medical Devices, Inc) were used, but later were changed to Hydromark clips, which have better visualization capacities under ultrasound at least 6 months after insertion.

Targeted axillary ultrasound and FNA was carried out by three general surgeons with the specialization in breast surgery, who have successfully passed EBSQ (European Breast Surgery Qualification) exam and have underwent National Ultrasound method postgraduate courses. In all cases sampling was done by the same physician before and after PST. All samples were reviewed by one cytologist experienced in breast pathology.

cN stage was diagnosed on the basis of pretreatment imaging (ultrasound and CT) data. If 1–3 involved axillary nodes were identified – cN1 stage was classified. If more than 3 metastatic lymph nodes were visualized in axilla, then cN2 stage was set. If infra/supraclavicular lymph node metastasis or more than 10 affected axillary nodes were detected, then cN3 stage was diagnosed. Frequency of cN1, cN2 and cN3 stage was observed in 58 (55 %), 23 (22 %) and 25 (23 %) cases respectively.

After PST restaging of axilla was performed with ultrasound guided FNAC of the marked and/or the most suspicious axillary node. Nondiagnostic cases were not included. In 10/106(9 %) cases, deviation from study protocol took place as core biopsy technique was used instead of FNAC.

Modified algorithm of Netherlands cancer institute/Antoni van Levenhoek hospital (NCI/AVL) was followed to decide on axilla conserving surgery (ACS) versus ALND as described by Koolen [4]. 72/106 axilla conserving surgeries (ACS) and 34/106 ALND were performed. Number of examined nodes in ACS group was 1–18, on average 6 and in ALND group 2–30, on average 13. Difference between retrieved nodes in ACS and ALND was statistically significant (*p* < 0.05).

In order to assess diagnostic value of ultrasound guided FNAC, FNR and FPR as well sensitivity, specificity, positive predictive value (PPV), negative predictive value (NPV) and accuracy were calculated. Statistical analyses were performed using Medcalc, easy-to-use software and MS Excel 2010. Fisher’s exact test was used for comparison of the results between groups.

## Results

Overall nodal pCR was observed in 41/106 (39 %) cases: Luminal − 19/64 (30 %); HER2 positive – 11/18 (61 %); TN – 11/24 (46 %). Distribution of nodal pCR in BRCA1/2 positive and negative subsets is -9/18(50 %) and 32/88 (36 %) respectively.

After PST FNAC revealed residual nodal cancer in 39/106 (37 %) cases, but in 67/106 (63 %) no malignancy was detected. In final surgical pathology in 65/106 (61 %) cases malignant cells persisted in axillary lymph nodes, but in 41/106 (39 %) cases no nodal tumor was detected after PST.

In 36/39 (92 %) cases, which had positive FNCA after PST, metastasis in axillary lymph nodes were revealed in the pathology examination of surgery specimen - ypN1mi, ypN1, ypN2 and ypN3 stage was detected in 1, 18, 11 and 6 cases respectively. Overall FPR of FNAC after PST is 3/39 (8 %). In contrary, only 38/67(57 %) cases with negative lymph node FNAC after PST, turned out to be without nodal involvement in surgery specimen examination. 29 false negative cases in final pathology revealed ypNmi, ypN1, ypN2, ypN3 nodal status in 7, 18, 3 and 1 case respectively. Overall FNR of FNCA after PST is 29/67 (43 %). FNAC was able to predict nodal response to PST correctly in 74/106 (70 %) of cases.

Further subgroup analysis revealed that in case of *BRCA1/2* positive breast cancers FPR was 0 % (0/7) and FNR was 18 % (2/11). In this subset FNAC was able to predict response to PST correctly in 16/18 (89 %) cases. Comparison of FNR between BRCA1/2 positive and BRCA1/2 negative subsets using Fisher’s exact test revealed statistical difference very close to significant, *p* value = 0,051502, but difference between FNR in BRCA1/2 positive and Luminal subsets was statistically significant, *p* = 027096.

Accuracy of FNAC test in TN, HER2 positive, Luminal and BRCA1/2 negative subgroups is 79 %, 78 %, 64 and 66 % respectively. Detailed results of FPR, FNR, sensitivity, specificity, positive and negative predictive value as well accuracy of the test in different subgroups see in Table 1.

**Table 1:** FPR, FNR, sensitivity, specificity, positive and negative predictive value as well accuracy of the FNAC test

	Overall	BRCA	Non BRCA	TN	HER2+	Luminal
FPR%	8 (3/39)	0 (0/7)	9 (3/32)	0 (0/8)	0 (0/3)	11 (3/28)
FNR%	43 (29/67)	18 (2/11)	48 (27/56)	31 (5/16)	27 (4/15)	56 (20/36)
Sensitivity%	55.38	77.78	51.79	61.54	42.86	55.56
Specificity%	92.68	100	90.62	100	100	84.21
Positive Predictive Value%	92.31	100	90.63	100	100	89.29
Negative Predictive Value%	56.72	81.82	51.79	68.75	73.33	44.44
Accuracy%	69.81	88.89	65.91	79.17	77.78	64.06

## Discussion

Axillary pCR after PST is frequent event in case of breast cancer [1–4]. Also, in our cohort we report 39 % of axillary pCR. Therefore, it is very important to perform appropriate restaging in node positive breast cancers after PST to avoid unnecessary ALND. One has to take in to account that there is no pertuzumab approved for PST in nonmetastatic setting in our country and with dual HER2 blockade pCR rate is expected to be even higher.

ACS, including clipped node biopsy alone or in combination with SNB has very low FNR [4, 9] and is very good approach to restage the axilla after PST, however in large proportion of node positive cases, especially cN2 and cN3 stage, still considerable residual nodal involvement remains, which requires ALND. One solution is frozen section, but it has several disadvantages: potentially prolonged surgery time and rather high FNR – 33 % [13]. It means that considerable proportion of cases potentially could require repeated surgery to perform ALND and it is not ideal management neither from patient nor hospital perspective. Therefore, it is important to have accurate diagnostic tools for axilla restaging preoperatively.

There are data on effectiveness of axillary ultrasound and FNAC to evaluate nodal status prior breast cancer treatment as well evaluation of ultrasound method alone after PST [14, 15]. However to the best of our knowledge there are only two small series (including our earlier one), which are reporting on axillary lymph node FNAC accuracy after PST [11, 12]. In our present study false negative rate of FNCA after PST is 43 %, sensitivity – 55.38 % and negative predictive value – 56.72 %. False positive rate is 8 % and specificity of the test is 92.68 %. Accuracy of test is 69.81 %. Obtained data are similar to those reported by Caudle et al.: sensitivity of 42.4 %, specificity of 100 %, and negative predictive value of 40.6 %. In the studies, which have evaluated the diagnostic value of axillary FNAC prior to PST, reported sensitivity, specificity and accuracy lies between 41 and 80 %, 96-100 % and 70–89 % respectively, but methodological differences exist [16–22]. We can conclude that in our study sensitivity of the FNAC is close to lowest range reported, but specificity and accuracy is clearly lower. One can conclude that accuracy of axillary FNAC after PST is somewhat lower than prior to PST. Low sensitivity of axillary FNAC together with high FNR in this setting precludes the omission of axillary surgery in spite of negative cytology. However, low FPR and specificity of the test is high enough to streamline surgical care with ALND for node-positive patients with considerable residual cancer burden expected. This approach has a potential to avoid frozen section and repeated axillary surgery in particular cases.

There are also report that sensitivity and specificity of axillary ultrasound alone after PST are 55 and 88 %, respectively, which is very close to results of our research [23–25]. However pathological confirmation of ycN stage is very important in surgical decision making especially taking in to account that ultrasound method is highly operator dependent.

In spite of high overall FNR, it is considerably lower in BRCA1/2 positive subgroup in comparison to BRCA1/2 negative subgroup (18 % vs. 48 %). This difference is very close to reach statistical significance and difference between FNR in BRCA1/2 positive and Luminal subgroups (18 % vs. 56 %) is statistically significant. Reasons for this potential finding are not completely clear, but could be related to the more homogenous patterns of nodal response to PST in case of BRCA1/2 carriers. However larger numbers are required to confirm this finding and potential for omission of axillary surgery in this subgroup on the basis of percutaneous biopsy only.

Like other studies we report significantly lower axillary pCR rate in Luminal subtype breast cancers [1]. Lobular breast cancers have extremely low rates of pCR after PST, but there are only 5/106 lobular cancer cases in our cohort and we are not into position to make any statements on this [26].

One of the potential biases of the study is inclusion of small number of core needle biopsy cases as well. For a short period of time it was allowed for responsible surgeon to choose between FNAC or core needle biopsy. However, according to literature data there is no considerable diagnostic value differences, but we presume that CNB is associated with smaller percentage of uninformative samples and repeated procedures respectively [27].

Another potential cause of bias - interobserver variability should be mentioned [28].

Present literature evidence continues to recommend FNAC as the most sensitive screening for breast cancer metastases in the axillary region. However, one should also mention innovative vacuum assisted breast biopsy (VABB) technologies, which could be considered in future studies. To the best of our knowledge there are no literature date on the use of this method to evaluate axillary lymph nodes after PST [29].

There are also number of calculation tools available to predict status of sentinel node in case of breast cancer with clinically negative axilla. However, as showed by experimental results by Fanizzi et al., CancerMath is not particularly suitable for use as a support instrument for the prediction of metastatic lymph nodes on clinically negative patients. And again, it should be emphasized that those tools are not validated in patients after PST, which is the target group of our study [30].

In our research we used only Gray-scale ultrasound. To improve gray-scale ultrasound results, some of the authors offer to use contrast-enhanced ultrasound (CEUS), elastography or colour Doppler. A systematic review and meta-analysis shows CEUS-guided core biopsy sensitivity 54 % and specificity 100 % in cases with normal axillary gray-scale ultrasound [31].

In another study grey-scale ultrasound was compared to elastography. Sensitivity and specificity for grey-scale ultrasound in detecting malignant nodes (defined by a cortical thickness > 3 mm) were 40 and 97 %. Sensitivity and specificity for elastography were 60 and 80 %. When grey-scale ultrasound and elastography were combined, the sensitivity and specificity rose to 73 and 99 %, respectively [32].

In spite of controversial reports, there are number of additional tools to be considered for future studies to improve axillary restaging after PST [33].

## Conclusions

FNAC after PST has low FPR and is useful to predict residual axillary disease and to streamline surgical decision making regarding ALND both in *BRCA1/2* positive and negative subgroups. FNR is high in overall cohort and FNAC alone are not able to predict ypCR and omission of further axillary surgery. However, FNAC performance in *BRCA1/2* positive subgroup is more promising and further research with larger number of cases is necessary to confirm the results.

## Data Availability

The datasets generated during and/or analyzed during the current study are available from the corresponding author on reasonable request.

## Abbreviations

FNAC: Fine needle aspiration cytology
PST: Preoperative systemic therapy
ALND: Axillary lymph node dissection
ACS: Axilla conserving surgery
FPR: False Positive Rate
FNR: False Negative Rate
pCR: Pathological complete response
NAC: Neoadjuvant chemotherapy
NCA/AVL: Netherlands cancer institute/Antoni van Levenhoek hospital
TN: Triple negative
PPV: Positive predictive value
NPV: Negative predictive value
